# Genomic signals of local adaptation in *Picea crassifolia*

**DOI:** 10.1186/s12870-023-04539-7

**Published:** 2023-11-03

**Authors:** Shuo Feng, Erning Xi, Wei Wan, Dafu Ru

**Affiliations:** 1 State Key Laboratory of Plateau Ecology and Agriculture, Qinghai University, Xining, 810016 People’s Republic of China; 2 State Key Laboratory of Herbage Improvement and Grassland Agro-Ecosystem, College of Ecology, Lanzhou University, Lanzhou, 730000 People’s Republic of China

**Keywords:** Global climate change, Transcriptome, Genetic structure, Redundancy analysis

## Abstract

**Background:**

Global climate change poses a grave threat to biodiversity and underscores the importance of identifying the genes and corresponding environmental factors involved in the adaptation of tree species for the purposes of conservation and forestry. This holds particularly true for spruce species, given their pivotal role as key constituents of the montane, boreal, and sub-alpine forests in the Northern Hemisphere.

**Results:**

Here, we used transcriptomes, species occurrence records, and environmental data to investigate the spatial genetic distribution of and the climate-associated genetic variation in *Picea crassifolia*. Our comprehensive analysis employing ADMIXTURE, principal component analysis (PCA) and phylogenetic methodologies showed that the species has a complex population structure with obvious differentiation among populations in different regions. Concurrently, our investigations into isolation by distance (IBD), isolation by environment (IBE), and niche differentiation among populations collectively suggests that local adaptations are driven by environmental heterogeneity. By integrating population genomics and environmental data using redundancy analysis (RDA), we identified a set of climate-associated single-nucleotide polymorphisms (SNPs) and showed that environmental isolation had a more significant impact than geographic isolation in promoting genetic differentiation. We also found that the candidate genes associated with altitude, temperature seasonality (Bio4) and precipitation in the wettest month (Bio13) may be useful for forest tree breeding.

**Conclusions:**

Our findings deepen our understanding of how species respond to climate change and highlight the importance of integrating genomic and environmental data in untangling local adaptations.

**Supplementary Information:**

The online version contains supplementary material available at 10.1186/s12870-023-04539-7.

## Introduction

In recent years, there has been increased interest in the effects of natural selection on local populations, particularly in the context of climate change and its potential impacts on biodiversity [1–3]. Across their geographic range, populations are subject to varying natural selection pressures due to environmental heterogeneity, thus resulting in local adaptation [4–7]. The phenomenon of local adaptation is particularly prevalent in forest trees, which live in one location for a long period of time, relative to animal species and herbaceous plants [8, 9]. However, their sessile nature, along with long generation times and slow reproduction rates, poses a significant challenge in keeping up with the rapid climate-induced changes that are likely to occur within the lifespan of an individual, leaving them susceptible to maladaptation [10–12]. Forest trees are important for absorbing and storing carbon dioxide, which helps to reduce greenhouse gas concentrations in the atmosphere and to mitigate the impacts of climate change [13, 14]. Therefore, identifying the genes and corresponding environmental factors involved in adaptation can provide crucial insights into genetic variation and aid the development of informed conservation and management strategies to further mitigate the impacts of climate change [15–17].

Conventional ways for studying local adaptation, such as reciprocal transplant or common garden experiments [18–20], pose significant challenges for many wild non-model organisms due to factors such as long generation time, difficulty in obtaining fitness-related phenotypic traits and experimental limitations [14]. However, advances in cost-effective next-generation sequencing technologies now enable the investigation of a vast number of loci, thus providing unprecedented insights into molecular adaptation across one species' distribution range [21–23]. The use of whole genome SNP data for studying natural populations has been proven to be reliable and powerful, and SNP identification and genotyping is now routine [24, 25]. Genotype-environmental association (GEA) approaches, which identify SNPs exhibiting elevated differentiation within environments and significant correlations between SNPs and environmental variables [26], are becoming more popular for detecting loci associated with climate adaptation [27–29]. The identification of climate-associated genetic variation not only helps solve fine-scale patterns of local adaptation but also deepens our understanding of how species respond to climate change [15]. Thus, GEA has emerged as a particularly useful approach for elucidating the genetic mechanisms underlying unique adaptations and responses to environmental stressors [10, 30, 31]. The genus *Picea* is a prime example of a dominant tree species found mainly in the northern temperate forest ecosystems of Asia, North America, and Europe [32, 33].

In this study, we focused on *Picea crassifolia*, an endemic, ecologically important tree species in China, to investigate its local adaptation and potential response to climate change. *P. crassifolia* is a highly-valued afforestation tree in northwest China due to its superior wood quality, serving as a source of wood for bridges, furniture and papermaking. *P. crassifolia* thrives at elevations ranging from 1,750 to 3,100 m, inhabiting mountainous areas characterized by overcast and semi-overcast slopes, as well as wet valley. While past studies have focused on the speciation between *P. crassifolia* and closely-related species, little is known about the species' adaptation to climate change and how its distribution may change in the future [34, 35].

To investigate the spatial pattern of genetic variation and the underlying genetic mechanisms of local adaptations in *P. crassifolia*, we conducted a transcriptome analysis on 82 individuals derived from 16 populations of this species across Gansu Province, Qinghai Province, and their surrounding areas in China. The aims of this study were (i) to examine genetic differentiation and population structure in *P. crassifolia*, (ii) to assess the respective influence of environmental and geographic factors on inter-population genetic differentiation while identifying key environmental variables that drive local adaptation, and (iii) to quantify the future potential distribution of this species under projected future climate scenarios.

## Methods

### Data preparation

**Fig. 1:**
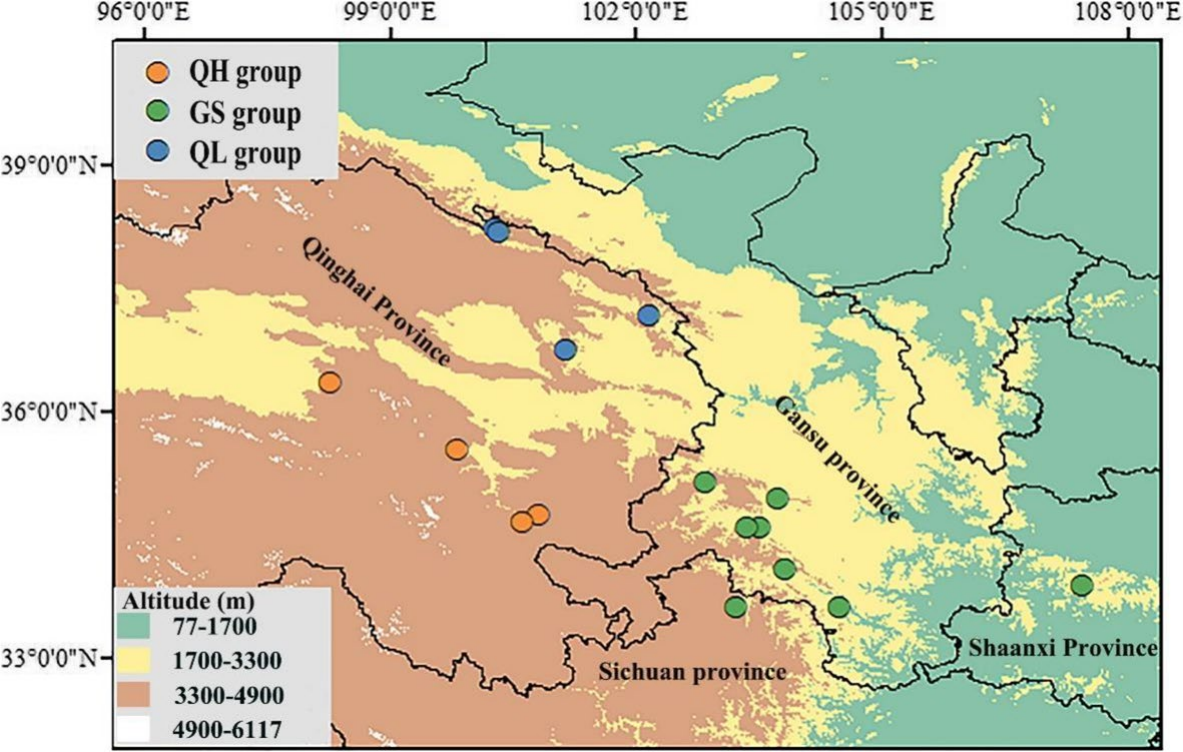
Geographic distribution of sampled *Picea crassifolia* populations. Individuals in the GS, QL and QH groups are distributed in Gansu Province and nearby areas, the Qilian Mountains and Qinghai Province, respectively. The map was downloaded from the DataV.GeoAtlas (http://datav.aliyun.com/portal/school/atlas/area_selector) and converted from json format to shp format in mapshaper (https://mapshaper.org/), finally constructed using the ArcGIS v10.7

Primary raw RNA-seq data encompassing 82 individuals from 16 populations of *P. crassifolia* was acquired through the NCBI database (NCBI BioProject Accession ID PRJNA846694). Additionally, we obtained the same data for a *P. wilsonii* individual from NCBI (NCBI BioProject Accession ID PRJNA401149) to serve as an outgroup. Each population consisted of a range of 3–10 individuals, and detailed information regarding RNA extraction, library construction and sequencing protocols can be found in the works of Feng et al. (2023) and Ru et al. (2018). The locations of the 16 populations are visually represented in Fig. 1 and Table 1.

**Table 1:** Population locations and sample sizes of 16 populations of *Picea crassifolia*

Population	Number of individuals	Location	Longitude (E)	Latitude (N)	Altitude (m)
GS	34	-	-	-	-
GGD	5	Diebu County, Gansu Province	103.8102	34.0567	2920.00
GGX	4	Xiahe County, Gansu Province	102.8369	35.1217	2740.00
GGZ	4	Zhouqu County, Gansu Province	104.4835	33.6051	1863.00
GJT	4	Taibai County, Shanxi Province	107.4502	33.8736	1981.00
GGH	4	Zhuoni County, Gansu Province	103.5008	34.5772	2750.00
GNZ	5	Lintan County, Gansu Province	103.7333	34.9300	2820.00
GGN	5	Zhuoni County, Gansu Province	103.3397	34.5678	2840.00
SAR	3	Ruoergai County, Sichuan Province	103.2033	33.6108	3250.00
QL	23	-	-	-	-
HZQ	6	Qilian County, Qinghai Province	100.2697	38.2261	3110.00
BZQ	10	Qilian County, Qinghai Province	100.3078	38.1735	2864.63
HZM	4	Menyuan Hui Autonomous County, Qinghai Province	102.1511	37.1725	2600.00
XNH	3	Huangyuan County, Qinghai Province	101.1411	36.7433	2870.00
QH	25	-	-	-	-
QHT	6	Tongde County, Qinghai Province	100.7958	34.7222	3410.00
QHM	7	Maqin County, Qinghai Province	100.6097	34.6480	3462.27
QHG	6	Dulan County, Qinghai Province	98.2475	36.3392	3580.00
QHX	6	Xinghai County, Qinghai Province	99.8100	35.5192	3580.00

*Abbreviations*: *GS P. crassifolia* populations from Gansu Province and nearby areas, *QL P. crassifolia* populations from the Qilian Mountains, *QH P. crassifolia* populations from Qinghai Province

### Read mapping and SNP calling

The paired-end raw reads were filtered and trimmed with fastp v0.20.0 [36]. This involved eliminating low-quality bases (Phred quality < 15), discarding reads with more than 5 N-bases or that exceeded 40 percent of low-quality bases, trimming ployX and ployG tails, removing the first 10 bases from both read1 and read2, and correcting bases in overlapping regions. After these trimmings and corrections, reads longer than 100bp were kept. The resulting clean reads from each individual were mapped to the reference transcriptome of *Picea abies* [37] using the BWA-MEM algorithm of BWA v0.7.17 [38] with default settings. Duplicate reads were flagged using the “MarkDuplicates” functionality of Picard-Tools v2.21.5 (http://broadinstitute.github.io/picard/) and were excluded from downstream analysis. Indels were detected and realigned using the “RealignerTargetCreator” and “IndelRealigner” tools from GenomeAnalysisTK v3.8 [39].

For variant calling, we utilized the “mpileup” command from SAMtools v0.1.19 [40], incorporating uniquely mapped reads, with parameters set as “-q 20 -Q 20 -t AD, ADF, ADR, DP, SP”. It is important to note that the raw variant sites may contain a significant number of false positives. To identify high-confidence SNPs, we employed the following filtering criteria: mapping quality > 30, genotyping rate > 50%, mapping depth > 10, minor allele frequency (MAF) > 0.05, SNPs must be biallelic and lack INDELs within a 5 bp window.

### Genetic structure and genetic diversity

The best nucleotide substitution model was determined using ModelFinder [41] and implemented in IQ-TREE v2.0.3 (http://www.iqtree.org), employing the Akaike information criterion (AIC). GTR + F + R3 was identified as the best-fitting model. Subsequently a maximum likelihood (ML) tree of 82 individuals was constructed using IQ-TREE v2.0.3 [42] with 1, 500 ultrafast bootstrap, a 1,500 SH-aLRT test, and *P. wilsonii* was set as an outgroup based on SNP data set.

To identify genetic clusters, a model-based genetic clustering analysis was conducted using ADMIXTURE v1.3.0 [43]. The SNP data set was preprocessed by converting it and removing linkage disequilibrium sites using VCFtools v0.1.14 [44] and PLINK v1.90 [45], with parameter “—plink” and “–indep-pairwise 50 5 0.2”, respectively. Cluster numbers (K) were set from 2 to 10, with 100 replicates per value. The optimal number of clusters was determined based on the cross-validation error (CV error) estimated for each cluster, and the K-value with the lowest CV error was selected. Subsequently, PCA was performed using PLINK v1.90 [45].

Using VCFtools v0.1.14 [44], we calculated the nucleotide diversity (*π*) in non-overlapping 100 bp windows. Pairwise genetic differentiation (*F*_ST_) per locus was calculated using VCFtools v0.1.14 [44] to estimate genetic differentiation between populations.

### Environmental variable and species occurrences selection

To investigate the local adaptation of *P. crassifolia*, we collected 56 environmental variables (including 19 bioclimatic variables, 12 solar radiation variables, 12 water vapour pressure variables, 12 wind speed variables and one topographic variable). All 55 environmental variables (averaged over the period from 1970–2000) and the topographic variable (altitude) were downloaded from Worldclim v2.1 (https://www.worldclim.org) at a spatial resolution of 30 arc-seconds. Species occurrences data were obtained from the Chinese Virtual Herbarium (CVH, http://www.cvh.ac.cn/), the Global Biodiversity Information Facility (GBIF, http://www.gbif.org) and field surveys. To avoid overfitting the niche models due to spatial autocorrelation of records [46], we rarefied occurrence records with the SDM toolbox [47] in ArcGIS v10.7 and only kept one record per 5-km radius. Finally, 56 environmental variables from 85 localities (covering 16 population locations and 69 species occurrences) were extracted using our custom R script (Additional file 1).

### Ecological niche divergence analysis

Multicollinearity was avoided by examining the Pearson correlation coefficients (*r*) among 56 environmental variables from 85 localities and eliminating one variable from each pair where |*r|*> 0.7. Consequently, 6 final environmental variables were selected, including Bio3 (isothermality), Bio4 (temperature seasonality), Bio14 (precipitation in the driest month), Bio15 (precipitation seasonality), Wind8 (wind speed in August) and Alt (altitude).

Next, the maximum entropy approach was implemented in MAXENT v3.4.4 [48] to determine potential distributions of the three groups (QL, QH, and GS) of *P. crassifolia*. A niche comparison was conducted using the niche overlap tool in ENMTools v1.4.4 [49] to quantify potential niche divergence between pairs of the three groups. Two test statistics were calculated, Schoener's *D* and standardized Hellinger distance (*I*). Both *D* and *I* had values ranging from 0 (no overlap) to 1 (complete overlap). The niche identity test was used to determine whether ecological niche modeling habitat suitability scores generated by the pairs of the three groups demonstrated statistically significant divergence from each other. The null distribution was generated by repeating the randomization procedure 100 times and comparing observed niche overlap values with the null distribution using a one-tailed test.

Further, ecological niche modeling was utilized to predict potential distributions for all individuals both today and in the future (2050, an average of predicted variables from 2041–2060). To predict future distribution, future climate layers from the SSP-126 and SSP-585 scenarios under the Sixth Phase of the Coupled Model Intercomparison Project (CMIP6) were downloaded from the WorldClim v2.1 (https://www.worldclim.org) database at a resolution of 30 arc-seconds.

Future climate models included only 19 bioclimatic variables. Therefore, for both current and future ecological niche modeling, five environmental variables present in both present and future models (except for Wind8) were selected and analyzed.

### Effects of IBD and IBE on genetic structure

To illustrate the influence of IBD and IBE on genetic composition, we calculated the relationship between environmental distance, geographic distance and genetic distance. To address multicollinearity, we assessed the pairwise Pearson correlation coefficients (*r*) among the 56 environmental variables from our 16 sampling sites, eliminating one variable in each pair where |*r*|> 0.7. To calculate pairwise geographic distances, we utilized the geographic coordinates of the sampling sites and employed the Geosphere v1.5–16 R package (https://github.com/rspatial/geosphere).

To calculate environmental distance (Euclidean distance) among the 16 populations, we employed the retained environmental variables and utilized the “dist” function in R software. We use Arlequin v3.5 [50] to calculate the genetic distance among populations. Subsequently. The partial Mantel test was used to detect significance between geographic/environmental distances and genetic distances with 9, 999 permutations in the Vegan v2.6–2 R package (https://github.com/vegandevs/vegan).

### Genotype-environment association analysis

Given that the genetic variation explained by environmental variables was greater than that explained by geographic variables (see results), only SNPs associated with environmental variables were identified. An RDA was conducted to identify candidate SNPs involved in local adaptations responding to the multivariate environment [26, 51]. Prior to running the RDA, we transformed longitude and latitude coordinates from each individual into Moran's eigenvector maps (dbMEM1 to dbMEM6) [26]. We calculated the eigenvalues for each constrained axis, and selected the first three axes for further investigation because they accounted for the majority of the genetic variation. SNPs were considered candidate sites if they were unique to one of the three groups and identified through RDA.

To determine the function of candidate loci, we searched for homologous genes in the non-redundant *Arabidopsis thaliana* protein database using DIAMOND v0.9.14 [52] with an E-value < 1.0 × 10^–5^. Gene ontology (GO) enrichment analyses were conducted with the ClusterProfiler v4.2.2 R package [53]. Benjamini–Hochberg FDR (false discovery rate) was used to correct *p*-values, and GO terms with corrected *p*-values ≤ 0.05 were considered significantly enriched.

## Results

### SNP calling, genetic diversity and population structure

The total raw data comprising 82 individuals amounted to 677.06 Gb, with an average of 8.26 Gb per individual. After filtering the raw data, 645.44 Gb of clean data was obtained. The reference transcriptome of *P. abies* was used as the reference for variant calling, and 120,855 high-quality SNPs were obtained after strict filtering of the initial variant loci.

**Fig. 2:**
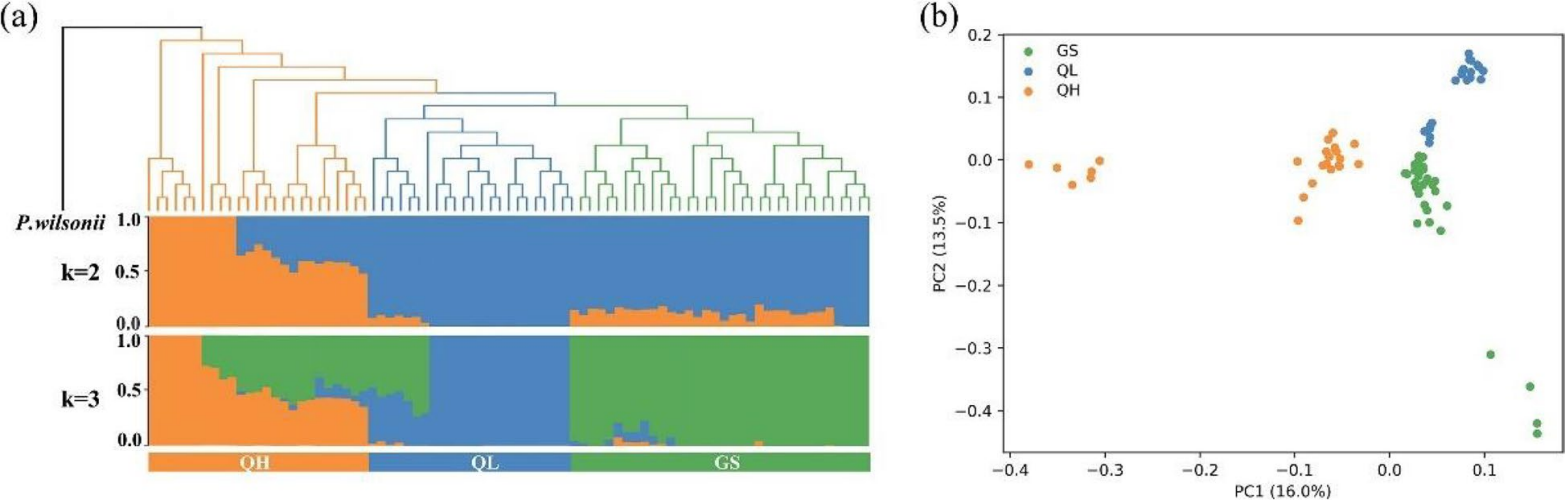
Genetic structure and phylogenetic relationships of the three *Picea crassifolia* groups (GS, QH and QL). **a** Admixture proportions of genetic clusters for each individual of the three groups and the resulting phylogenetic tree. The scenarios of K = 2 and K = 3 are shown. K = 2 has the best value according to cross-validation analysis. A maximum likelihood (ML) tree based on 120,855 SNPs with *Picea wilsonii* as the outgroup was constructed above the admixture result. **b** Principal component analysis (PCA) plot for the 82 *P. crassifolia* individuals based on the first two principal components

The phylogenetic analysis of these 120,855 SNPs loci revealed the presence of three lineages of *P. crassifolia* in Gansu and Qinghai provinces (Fig. 2a). The three lineages were labeled GS, QH and QL (Table 1). The QH lineage occupied the most basal position, followed by the GS + QL lineage. In addition, clustering analysis software ADMIXTURE was used to analyze the population structure of *P. crassifolia*. The results showed that the CV error was minimized at clustering number K = 2 (Additional file 2). Accordingly, the 16 *P. crassifolia* populations were divided into QH and GS + QL groups (Fig. 2a). When K = 3, CV error was the second lowest, and the QL group was separated from GS, aligning with the phylogenetic tree. PCA results demonstrated that the first principal component (PC1) explained 16.0% of the genetic variance, and segregated the 82 *P. crassifolia* individuals into QH and GS + QL group, which is consistent with the population clustering analysis at K = 2 (Fig. 2a, b). The second principal component (PC2) accounted for 13.5% of the genetic variance and differentiated the QL group from the GS group. Notably, the findings from clustering analysis, phylogenetic analysis, and PCA aligned with the geographic distribution of the sampling sites.

Furthermore, aside from calculating the nucleotide diversity (*π*) for the overall population and for each group, we also determined the number of SNPs exclusive to each group, the shared SNPs, and the *F*_ST_ values between groups. The *π* value at the species level was 0.003364, while the *π* of the three groups ranged from 0.003658 to 0.003843, with the highest in the QH group and the lowest in the GS group (Table 2). These values closely resemble those observed in other Pinaceae plants, such as *Pinus densata* (*π* = 0.0028), *Pinus yunnanensis* (*π* = 0.0024), *Picea purpurea* (*π* = 0.00392) and *Picea wilsoni* (*π* = 0.00392) [54, 55]. Among the three groups, the GS group (6,661) had the highest number of private SNPs, followed by the QH group (3,129), and the QL group (2,964) had the fewest private SNPs (Table 2). The number of shared SNPs between groups ranged from 89,453 (QL vs. QH) to 95,569 (GS vs. QH), and the *F*_ST_ values ranged from 0.013994 (QL vs. GS) to 0.023407 (QL vs. QH) (Table 2).

**Table 2:** Summary of genomic polymorphisms and variants in different *Picea crassifolia* groups

Parameter	All individuals	Groups			Between groups		
		QL	GS	QH	QL vs. GS	QL vs. QH	GS vs. QH
SNPs	120,855	101,374	111,267	102,052	-	-	-
Private SNPs	-	2964	6661	3129	-	-	-
*π*	0.003364	0.003819	0.003658	0.003843	-	-	-
Shared SNPs	-	-	-	-	95,031	89,453	95,569
*F*ST	-	-	-	-	0.013944	0.023407	0.016836

### Ecological niche divergence

**Fig. 3:**
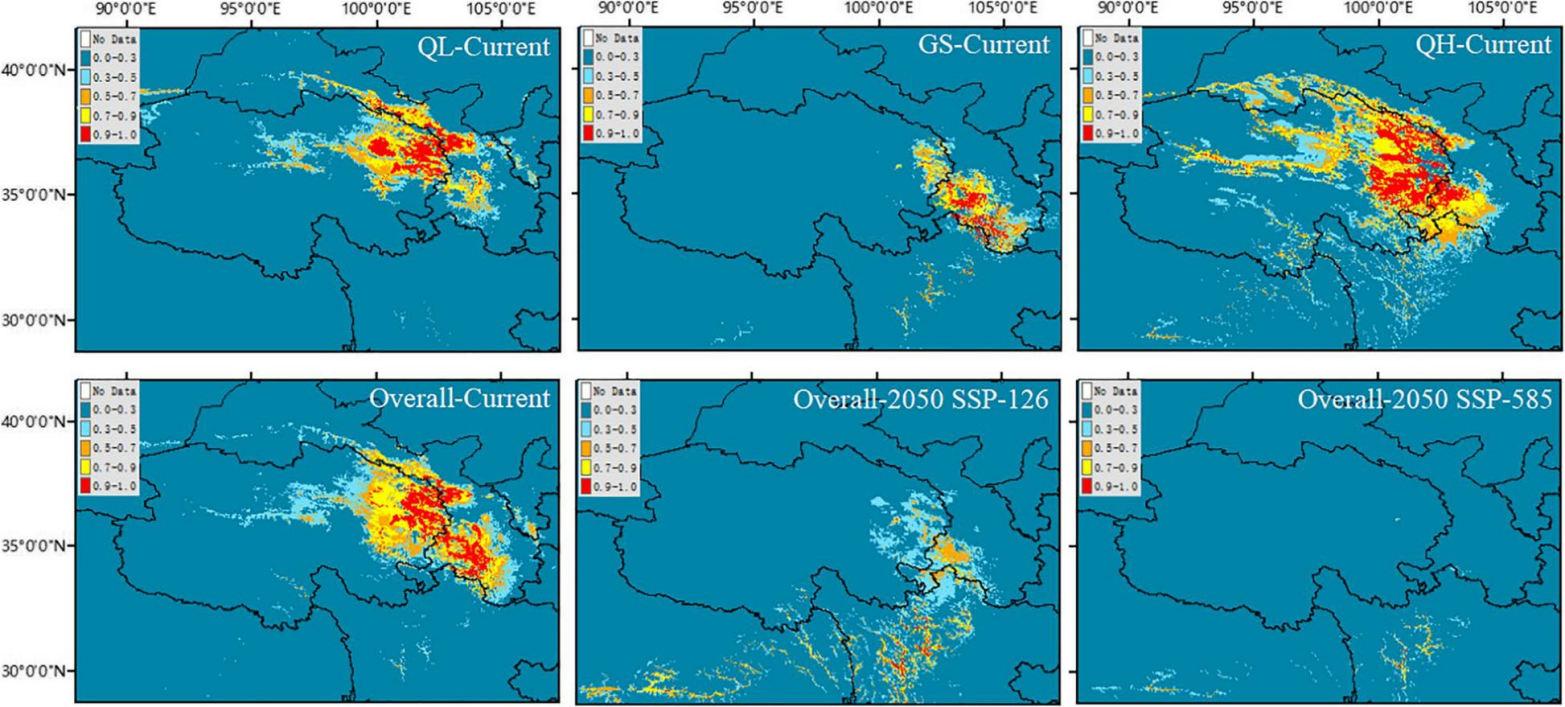
Potential distributions of *Picea crassifolia* groups (QL, GS, QH) for the present time (current), and overall *Picea crassifolia* for the present time (current) and two shared socioeconomic pathways (SSPs) in future (2050). The map was downloaded from the DataV.GeoAtlas (http://datav.aliyun.com/portal/school/atlas/area_selector) and converted from json format to shp format in mapshaper (https://mapshaper.org/), finally constructed using the ArcGIS v10.7

**Fig. 4:**
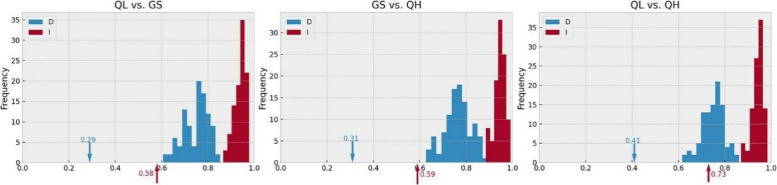
The niche differences between pairs of the three groups of *Picea crassifolia*. The bars represent Schoener's *D* and Hellinger's distance (*I*) with an identity test. Arrows indicate the values of *D* and *I* in maxent runs

We conducted ecological niche modeling for the three groups of *P. crassifolia*. A total of 85 occurrence records for *P. crassifolia* were obtained using our rigorous methods, with 20 records belonged to the QH group, 26 to the GS group, and 39 to the QL group (Additional file 1). Area under the receiver operating characteristic curve (AUC) values of all Maxent models exceeded 0.95, indicating that each model had high predictive performance. The current optimal ecological space of the QL and GS groups in the Maxent models is basically consistent with their present distributions. However, the optimal ecological space of the QH group is larger than its present distribution (Fig. 3). Observed Schoener's *D* and standardized Hellinger distances (*I*) generated by Maxent runs were below the critical values for null distributions concerning group pairs, indicating high niche differentiation in group pairs (Fig. 4).

Moreover, ecological niche modeling was performed on all *P. crassifolia* individuals to determine their potential distribution in the present and future. Under the current climate, the predicted distribution of *P. crassifolia* was basically consistent with the actual distribution (Fig. 3). However, when compared with the present distribution model, the *P. crassifolia* future distribution model exhibited reduction and migration due to a shift in suitable habitats. Notably, under the most extreme climate scenario (SSP-585), which predicts a more pronounced warming trend, the potentially suitable habitat for *P. crassifolia* faces even greater threats (Fig. 3).

### Effects of IDB and IBE on genetic structure

**Fig. 5:**
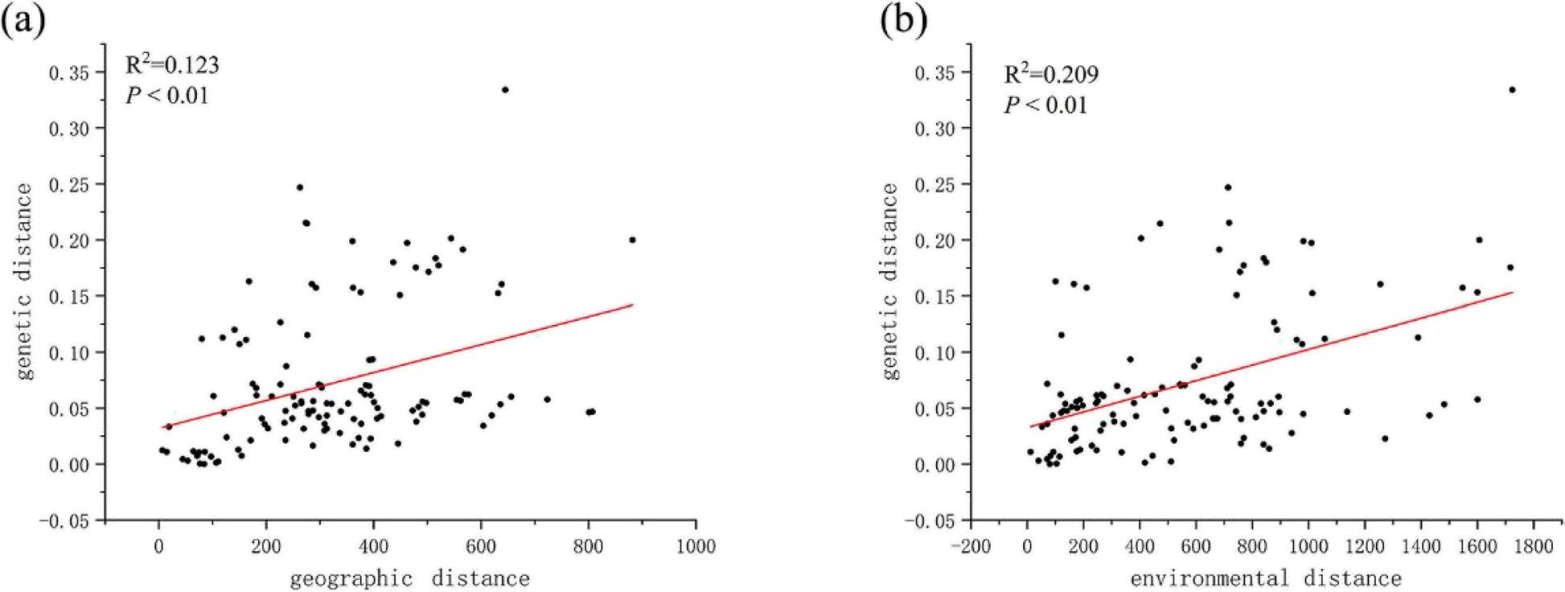
Effects of geographic and environmental variables on genetic structure of the *Picea crassifolia* population. The relationships are of genetic distance with (**a**) geographic distance and (**b**) environmental distance

To investigate the effects of IBE on genetic structure, we selected five environmental variables (Bio4: temperature seasonality, Bio14: precipitation in the driest month, Bio13: precipitation in the wettest month, Wind2: Wind speed in February and Alt: altitude) for further analyses. Subsequently, we used the partial Mantel test to examine the relationship between geographic/environmental distance and genetic distance. The results revealed a significant relationship between genetic distance and both geographic distance and environmental distance. Notably, environmental distance (*R*^*2*^ = 0.209, *p* < 0.01) exerted a stronger influence on genetic structure than did geographic distance (*R*^*2*^ = 0.123, *p* < 0.01) (Fig. 5). We further investigated the independent contribution of the environmental and geographic variables shown in Additional file 3 to genetic variation using partial RDA. As shown in Table 3, when controlling for environmental variables (partial model gen. ~ geo. | env.), geographic variables had a significant effect on genetic variation and explained 2.33% of genetic variation alone (adjusted *R*^*2*^ = 0.0233, *p* ≤ 0.001). Likewise, when controlling for geographic variables (partial model gen. ~ env. | geo.), environmental variables significantly influenced genetic variation, explained 3.41% of genetic variation alone (adjusted *R*^*2*^ = 0.0341, *p* ≤ 0.001). The combined effect of confounded geographic and environmental variables accounted for 0.86% of the genetic variation. The total genetic variation explained by the model incorporating both geographic and environmental variables (model gen. ~ geo. + env.) was 6.60%. Remarkably, environmental variables (RDA model gen. ~ env. adjusted *R*^*2*^ = 0.0427, *p* ≤ 0.001) explained a higher proportion of genetic variation compared to geographic variables (RDA model gen. ~ geo. adjusted *R*^*2*^ = 0.0320, *p* ≤ 0.001). In summary, both geographic and environmental variables significantly influenced the genetic differentiation of the *P. crassifolia* population. However, the impact of environmental variables was more pronounced than that of geographic variables.

**Table 3:** Redundancy analyses (RDA) to partition genetic variation (gen.) in *Picea crassifolia* into environment (env.), geography (geo.), and their joint effects

	Adjusted *R*^*2*^	F	*p*-value	df
RDA				
gen. ~ env	0.0427	1.7228	0.001	5
gen. ~ geo	0.0320	1.5349	0.001	5
partial RDA				
gen. ~ env. | geo	0.0341	1.5545	0.001	5
gen. ~ geo. | env	0.0233	1.3795	0.001	5
confounded	0.0086			
gen. ~ geo. + env	0.0660	1.5727	0.001	10

gen., SNP data matrix; env., five retained environmental variables; geo., five retained Moran’s eigenvector map variables; confounded, total of individual fractions confounded between various combinations of environment and geography

### Characterization of local adaption

**Fig. 6:**
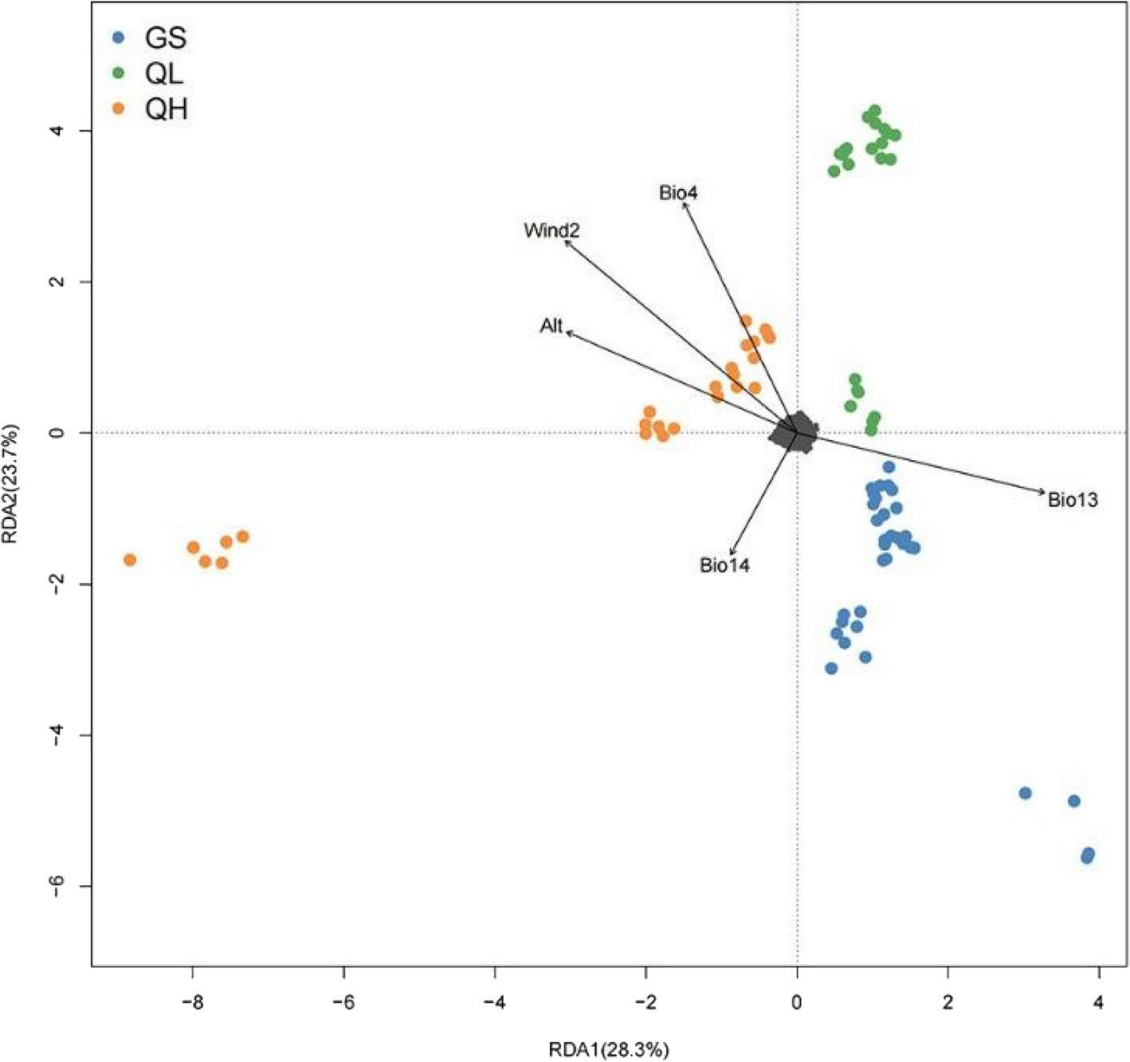
Redundancy analysis (RDA) plot for the 82 *Picea crassifolia* individuals based on the first two RDA axes

The adaptation of plants to the heterogeneous environment is mainly reflected in two aspects: phenotypic difference and genotypic difference. We found significant differences in needle morphology, including length and width, between groups inhabiting different geographical areas and habitats. This is one example of phenotypic evidence for local adaptation. It is worth noting that the variation in plant phenotypic traits predominantly arises from divergent selection pressures exerted by external environmental variables [56, 57]. To further study the association between genotype and environment, we used RDA, a multivariate ordination technique [26, 51], to explore the SNPs associated with environmental variables. The RDA results revealed a significant correlation (*p* ≤ 0.001) between the SNP matrix and environmental variables along the first three axes. Genetic variation was explained by RDA axes 1, 2, and 3 at rates of 28.3%, 23.7%, and 19.9%, respectively. RDA axis 1 divided 16 *P. crassifolia* populations into the QH group and the QL + GS group, and RDA axis 2 separated the QL group from the QL + GS group (Fig. 6). These results indicate that environmental variables play a key role in intraspecific differentiation among *P. crassifolia* populations. Alt, Bio13, and Wind2 had higher loads on RDA axis 1 than the other variables. Bio14 and Bio4 had high loads on RDA axis 2. This suggests that these environmental variables may play a crucial role in driving the adaptation of different *P. crassifolia* populations to their respective environments.

In our analysis, SNP loadings were calculated from the first three significant constrained axes (RDA axes 1, 2 and 3), and the histograms of the loadings on each RDA axis appeared to follow a relatively normal distribution (Additional file 4). As shown in the histograms, SNPs that loaded near the center of the distribution did not show a strong relationship with environmental predictors. Conversely, the SNPs located at the axis tails were more likely to undergo selection. With a cutoff of three standard deviations (*p* < 0.0027), we extracted SNPs loaded on the axis tails.

A total of 2,760 environment-associated SNPs were identified through RDA analysis. Among these SNPs, 2,009 were loaded on RDA axis 1469 were loaded on RDA axis 2, and 282 were loaded on RDA axis 3 (Additional file 5). Out of the 2,760 SNPs, 80 (residing in 73 genes) were private to the QL group, 18 (residing in 7 genes) were private to the GS group, and 840 (residing in 686 genes) were private to the QH group (Additional file 5). GO annotation was performed on 7 genes of the GS group, resulting in the annotation of 3 genes associated with catalytic activity (GO: 0003824), fatty acid biosynthetic process (GO: 0006633) and oxidoreductase activity (GO: 0016491), respectively. Furthermore, GO enrichment analysis was conducted on 80 genes in the QL group and 686 genes in the QH group. Among the 80 genes in the QL group, 13 genes exhibited significant enrichment. Specifically, 2 genes were assigned to the biological process category, with the association of the term “rRNA processing”, 2 genes were assigned to the cellular component category, with the association of the term “endoplasmic reticulum”, and 9 genes were assigned to the molecular function category, associated with terms such as “GTP binding”, “proton-transporting ATP synthase activity, rotational mechanism” and “GTPase activity” (Additional file 6). Among the 686 genes in the QH group, 34 genes showed significant enrichment. Of these, 9 genes were associated with biological processes, with “protein transport” being a significant associated term; 7 genes were associated with cell components, with the involvement of the “Golgi apparatus”; and 18 genes were associated with molecular functions such as “glycosyltransferase activity” and “GTPase activity” (Additional file 7).

## Discussion

Climate change is a significant global phenomenon that has been observed and that is expected to continue in the twenty-first century. Organisms have three possible responses to climate change: they can either disperse and change their range limits, adapt through phenotypic plasticity, or evolve genetically to match the new climatic conditions [58, 59]. In this study, we conducted a comprehensive analysis of transcriptome-based SNP variants for *P. crassifolia*. All our analyses confirmed the existence of three distinct genetic clusters, which was consistent with the results of a previous study [34]. Furthermore, our population genomic analyses indicated clear evidence of local adaptations associated with local environmental conditions among these three genetic clusters. The study also revealed a potential threat to the survival of *P. crassifolia* populations in the future. Our results demonstrate the complexity of local adaptation in *P. crassifolia* and emphasize the importance of considering multiple factors in conservation efforts aimed at preserving plant populations in the face of climate change.

### Intraspecific divergence

In our study, ADMIXTURE, PCA and phylogenetic tree results showed that the *P. crassifolia* population distributed in and around Gansu and Qinghai provinces can be divided into three genetic clusters (Figs. 1, and 2a, b). Interestingly, we observed little variation in nucleotide diversity (*π*) among the three *P. crassifolia* groups, though the cluster *π* values were notably higher than the species-level value (Table 2). This finding suggests that the genetic diversity within each group is relatively high and may be the result of long-term local adaptation or historical isolation and limited gene flow between populations [17, 60]. This conclusion is further corroborated by the results of ecological niche divergence, IBD and IBE, and RDA (Figs. 4, and 5; Table 3). The high nucleotide diversity observed within each group is promising for the species’ capacity to adapt to changing environmental conditions in the short-term because genetic diversity is crucial for a species' ability to adapt to novel environments [61–63]. Furthermore, our analysis revealed that the QL and GS groups had the lowest genetic differentiation, suggesting a relatively high level of gene flow between the two groups, possibly due to their close geographic proximity. Conversely, we observed the highest genetic differentiation between the QL and QH groups. This indicates a lower level of gene flow due to their significant geographic distance and distinct ecological niches (Fig. 4).

### Evidence for polygenic adaptation to climate

Geographic and environmental isolation can lead to genetic divergence [64] as populations adapt to their local environment and ecological niches. The role of environmental factors in driving genetic variation among populations is well established [65]. The ecological niche differentiation, partial Mantel test and partial RDA models all showed that the environmental factors played a major role in driving or maintaining genetic variation among populations. Specifically, the RDA analysis identified five environmental variables that could significantly predict genetic variation in populations, and these five explained 4.27% of genetic variation observed (Table 3).

Our study also identified private SNPs in each group, reflecting their local adaptation to various environmental factors. The presence of SNPs significantly associated with environmental variables highlights the complexity of local adaptations in response to different environmental conditions. We identified 18 SNPs in 7 genes that may have contributed to habitat adaptation in the GS group. The GO annotation of these genes revealed that three were related to catalytic activity, fatty acid biosynthetic process and oxidoreductase activity, respectively. Fatty acids are crucial components of plant membranes and play an important role in pollen wall formation [66–68]. Catalytic activity and oxidoreductase activity are closely related to metabolism and synthesis of chemical compounds within organisms. Moreover, it is noteworthy that the Annual Mean Temperature (Bio1) was higher in the GS group than in the other two groups (Additional file 1). This elevation in temperature corresponds to earlier flowering time, accelerated metabolism rate, and heightened enzyme activity [65, 69–71]. Consequently, the candidate genes identified within the GS group likely contributed to the species' adaptation to this higher temperature environment.

Within the QL group, we identified 73 candidate genes (containing 80 SNPs), and GO analysis showed that the most representative terms were related to GTP binding, rRNA processing, GTPase activity, and proton-transporting ATP synthase activity (Additional file 6). GTP and ATP are involved in cellular energy conversion, while GTP is also involved in gene translation and signal transduction [72, 73]. In terms of environmental variables, Temperature Seasonality (Bio4), Temperature Annual Range (Bio7) and Annual Precipitation (Bio12) within the QL group differed significantly from their counterparts in the GS group (Additional file 1). Furthermore, the environmental differences observed between the QL group and the QH group can be attributed mainly to variation in altitude (Additional file 1). In light of these environmental distinctions, it can be reasonably inferred that the candidate genes within the QL group are intricately linked to the adaptation of this species to the intricate and dynamic climate conditions encountered within its habitat.

In the QH group, we identified 686 candidate genes (containing 840 SNPs), and enrichment analysis showed that Golgi apparatus, glycosyltransferase activity, GTPase activity and protein transport were the most representative terms (Additional file 7). In addition, notable candidate genes related to chloroplasts and ubiquitin were also evident in this context (Additional file 7). The significance of these findings becomes apparent when considering their biological implications. The glycosylation reaction involving glycosyltransferase plays an important role in plant growth and metabolism regulation [74, 75]. These reactions also involve the Golgi apparatus and protein transport, thus influencing various aspects of plant physiology and development. Furthermore, the presence of candidate genes associated with chloroplasts is noteworthy due to their close association with photosynthesis, a fundamental process in plants. Lastly, the ubiquitin system is involved in adaptation to high altitudes [76]. It is imperative to underscore that the QH group's habitat is characterized by high altitudes, where strong ultraviolet radiation that can seriously affect plant photosynthesis is prevalent. Thus, these candidate genes may promote the adaptation of this species to the challenging environmental conditions associated with high altitudes. Furthermore, it is worth noting that the functions of most candidate genes remain unknown, yet they may play an essential role in the local adaptation of populations.

Significant differences in the number of candidate genes among the three groups are discernible, and this may be attributed to local adaptation, subsequently leading to significant distinctions in needle morphology. Compared to the other two groups, the GS group exhibited the highest Annual Mean Temperature (Bio1), the greatest Annual Precipitation (Bio12), and the smallest Temperature Annual Range (Bio7) (Additional file 1). Consequently it experienced relatively few external stressors, rendering most mutations neutral. Conversely, the QL and QH groups, with lower Annual Mean Temperature (Bio1), lower Annual Precipitation (Bio12), and broader Temperature Annual Range (Bio7) than the GS group, faced greater environmental stressors, resulting in more SNPs associated with these conditions. Notably, the QH group, while similar to the QL group in bioclimatic variables, thrived at higher altitudes (Additional file 1), which accounts for the discovery of the greatest number of private SNPs associated with environmental factors in this group. However, it is important to note that much of the observed genetic variation remains unexplained because (1) most SNPs are neutral; (2) the multivariate linear association model used in RDA has limitations; and (3) unmeasured environmental variables may also bear some influence. Further research is needed to fully understand the complex interplay between environmental factors and genetic variation in natural populations.

### Threats of climate change to this species and conservation implications

Forests play a crucial role in regulating global carbon and nitrogen cycles and are considered the global regulators of the world’s climate [77–79]. Disturbances in forest ecology can have adverse effects on the micro and macro-climates [80]. Climate change induces alterations in the typical ecosystem structure and function that can lead to disasters such as forest fires, droughts and pest outbreaks, and that can impact forest health and the livelihoods of forest-dependent communities [81, 82]. The results of the niche modeling were consistent with the actual distribution, except for the potential distribution of QH, which was broader than the actual distribution (Fig. 3; Additional file 1). The inconsistency between the model results and the actual distribution of QH may be due to the lack of species records, the geographic distance between existing records (Additional file 1), species extinction due to human factors, or unsurveyed areas that leave gaps in the dataset. However, the results of the niche models for all individuals of *P. crassifolia* suggest that suitable habitats will be reduced in the future (2050), relative to the current situation (Fig. 3). This implies that climate change poses a serious threat to the future distribution and survival of the species, a finding that is consistent with previous studies [83, 84].

Therefore, in concert with our study elucidating the local adaptation dynamics within this species, we encourage the implementation of conservation strategies aimed at protecting the genetic diversity of *P. crassifolia* due to its wide distribution range and obvious population structure. Effective conservation efforts for plant populations facing climate change are critical for maintaining genetic diversity, local adaptation, and gene flow [58, 85, 86]. Firstly, we found that the species has significant genetic differentiation and population structure among natural populations distributed in different regions (Fig. 2; Table 2). Through long-term interaction with their respective environments, different populations have forged genetic profiles that promote species adaptation to their local milieu. For example, the candidate genes within the QH group propelled the species toward acclimatization to high-altitude environs, while the candidate genes of the GS group promoted adaptation to elevated temperatures. Local adaptation and genetic diversity are essential for the survival and adaptation of species under future climate change. Considering the adaptability of different populations to their respective local environments, one of the most effective ways to protect this species is to maintain the existing local adaptions and genetic diversity of this species through in situ management and conservation [87–89]. Secondly, the various populations have distinct genotypes at loci pertinent to environmental adaptation, and geographic isolation prevents gene flow between these populations. Therefore, the prudent course of action involves the collection of seeds from natural populations located in different geographic regions to establish germplasm banks. Planting these seeds in nurseries can promote gene flow between populations and enhance species genetic diversity [88–92]. These conservation efforts will help ensure the survival of *P. crassifolia* and promote its adaptation to future climate change.

## Conclusion

Global warming and observed climatic changes are significant global phenomena that have affected and will continue to affect organisms worldwide. This study examined the adaptation of *P. crassifolia* to the climate using transcriptome-based SNP variants. The results show that 16 populations of the species can be divided into three groups, according to obvious genetic and niche differentiation, and that future climate change poses a serious threat to the distribution and survival of the species. Both environmental and geographic isolation were significantly associated with genetic differentiation among populations, but the contribution of environmental isolation was more significant than that of geographic isolation. We also identified key environmental variables and genes that drive local adaptation in each group. To preserve the genetic diversity of the species and to ensure its survival, conservation strategies such as in situ conservation and planting natural populations from different regions are recommended. Overall, the study results provide us with a clearer understanding of species adaptation to climate and have important implications for species improvement and conservation in the future.

### Supplementary Information

**Additional file 1. **Fifty-six environmental variables for 85 occurrence records (including 16 population locations) of *Picea crassifolia*.

**Additional file 2.** Cross-validation error plot of each K (K = 2 to 10).

**Additional file 3.** The significant environmental and geographic variables retained by the initial step-forward selection method.

**Additional file 4**. Histograms of SNPs loaded on the first three significant RDA axes.

**Additional file 5.** Environmental associated loci (EALs) identified using RDA.

**Additional file 6.** GO enrichment for 73 candidate genes for the QL group.

**Additional file 7.** GO enrichment for 686 candidate genes for the QH group.

## Data Availability

The raw sequence data analysed during the current study have been deposited in the Short Read Archive (SRA) of the NCBI under accession number PRJNA846694 and PRJNA401149.

## Abbreviations

RDA: Redundancy analysis
SNP: Single-nucleotide polymorphism
GEA: Genotype-environmental association
MAF: Minor allele frequency
AIC: Akaike information criterion
ML: Maximum likelihood
IBD: Isolation by distance
IBE: Isolation by environment
PCA: Principal component analysis
AUC: Area under the receiver operating characteristic curve
GO: Gene ontology
*F*_ST_: Pairwise genetic differentiation
*π*: Nucleotide diversity
