# Supplementary figures and images for: Genomic signals of local adaptation in Picea crassifolia
Source: BMC Plant Biol. 2023 Nov 3;23:534. doi: 10.1186/s12870-023-04539-7 (PMC10623705; doi:10.1186/s12870-023-04539-7)

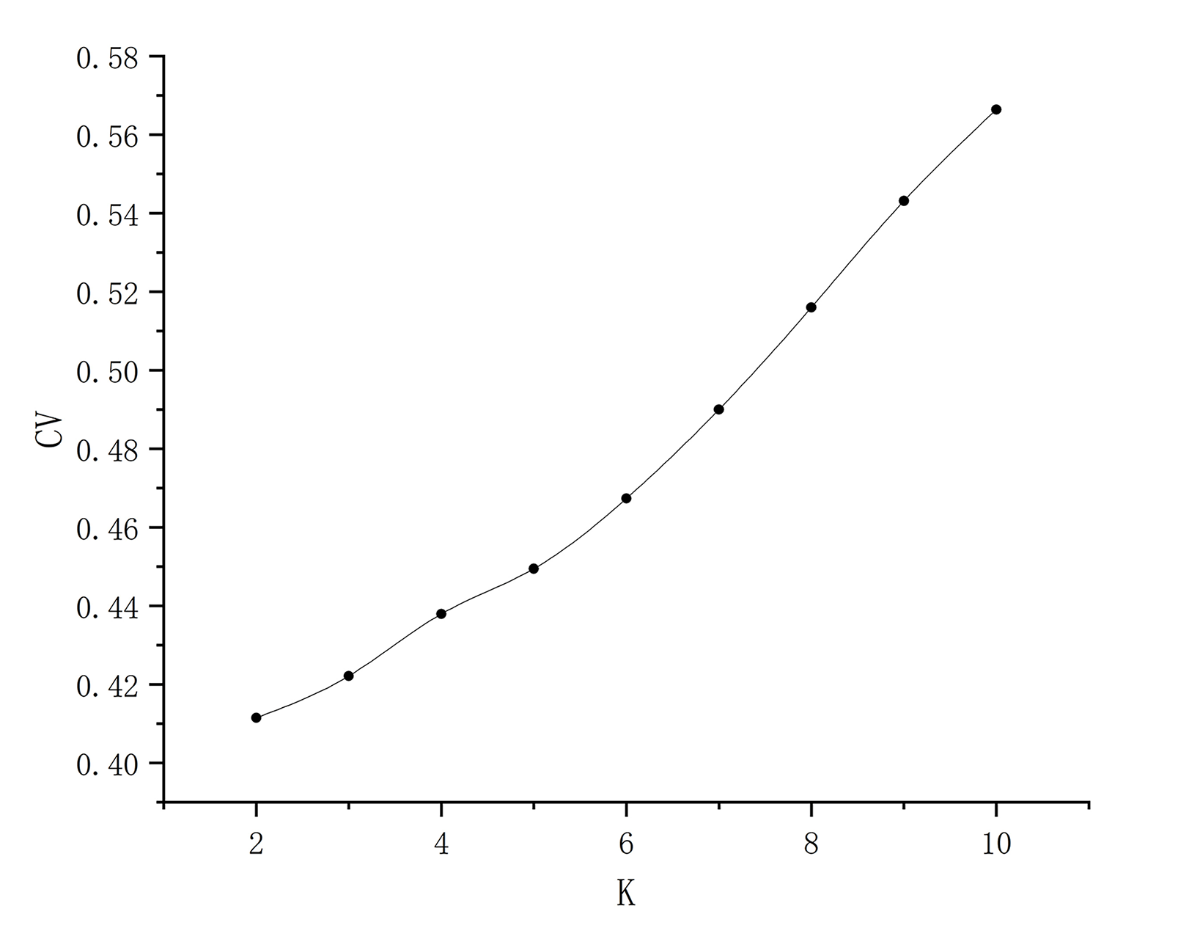


Additional file 2 | Cross-validation error plot of each K (K = 2 to 10)

Supplement: Supplementary file 2 — Additional file 2. Cross-validation error plot of each K (K = 2 to 10). [file 12870_2023_4539_MOESM2_ESM.docx]
