# Supplementary material for: Genomic signals of local adaptation in Picea crassifolia
Source: BMC Plant Biol. 2023 Nov 3;23:534. doi: 10.1186/s12870-023-04539-7 (PMC10623705; doi:10.1186/s12870-023-04539-7)
Supplement: Supplementary file 3 — Additional file 3. The significant environmental and geographic variables retained by the initial step-forward selection method. [file 12870_2023_4539_MOESM3_ESM.docx]

Additional file 3 | The significant environmental and geographic variables retained by the initial step-forward selection method

| **Variable** | **Adjusted *R*^2^** | ***F*** | ***p*-value** |
| --- | --- | --- | --- |
| Environmental variables | | | |
| Alt | 0.01623140 | 1.383463 | 0.001 |
| Bio4 | 0.02236267 | 1.860006 | 0.001 |
| Bio13 | 0.02278377 | 1.917029 | 0.001 |
| Bio14 | 0.01912791 | 1.622263 | 0.001 |
| Wind2 | 0.02782822 | 2.289984 | 0.001 |
| Geographic variables | | | |
| MEM1 | 0.02396642 | 1.964393 | 0.001 |
| MEM2 | 0.01780234 | 1.490385 | 0.001 |
| MEM3 | 0.01950957 | 1.611310 | 0.001 |
| MEM4 | 0.01897256 | 1.578430 | 0.001 |
| MEM5 | 0.01618367 | 1.361228 | 0.001 |
