# Supplementary material for: Genomic signals of local adaptation in Picea crassifolia
Source: BMC Plant Biol. 2023 Nov 3;23:534. doi: 10.1186/s12870-023-04539-7 (PMC10623705; doi:10.1186/s12870-023-04539-7)
Supplement: Supplementary file 4 — Additional file 4. Histograms of SNPs loaded on the first three significant RDA axes. [file 12870_2023_4539_MOESM4_ESM.docx]

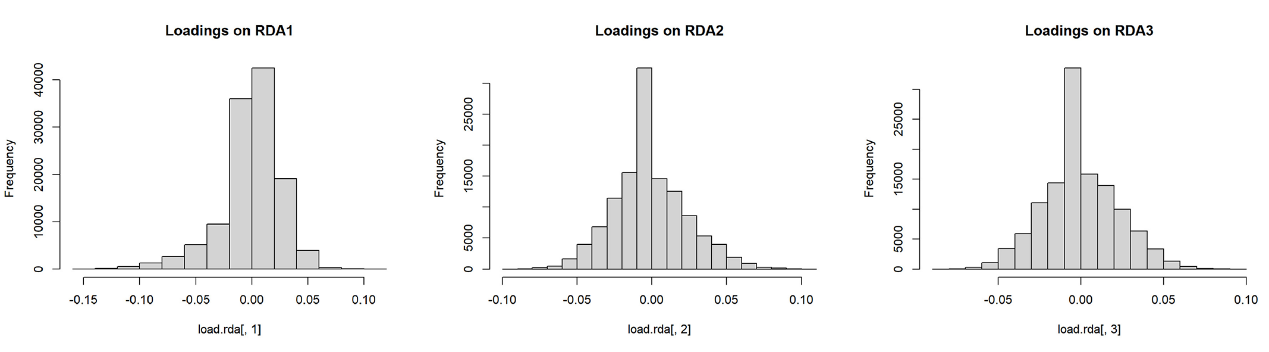


Additional file 4 | Histograms of SNPs loaded on the first three significant RDA axes. Null distributions are shown by grey bars. (SNPs loaded at the center of the distribution do not show a relationship with the environmental predictors, while those loaded on the tails are more likely to be under selection as a function of the predictors.)
